# Modeling depth from motion parallax with the motion/pursuit ratio

**DOI:** 10.3389/fpsyg.2014.01103

**Published:** 2014-10-06

**Authors:** Mark Nawrot, Michael Ratzlaff, Zachary Leonard, Keith Stroyan

**Affiliations:** ^1^Department of Psychology, Center for Visual and Cognitive Neuroscience, North Dakota State UniversityFargo, ND, USA; ^2^Math Department, University of IowaIowa City, IA, USA

**Keywords:** depth perception, motion parallax, pursuit eye movements, stereopsis, motion perception

## Abstract

The perception of unambiguous scaled depth from motion parallax relies on both retinal image motion and an extra-retinal pursuit eye movement signal. The motion/pursuit ratio represents a dynamic geometric model linking these two proximal cues to the ratio of depth to viewing distance. An important step in understanding the visual mechanisms serving the perception of depth from motion parallax is to determine the relationship between these stimulus parameters and empirically determined perceived depth magnitude. Observers compared perceived depth magnitude of dynamic motion parallax stimuli to static binocular disparity comparison stimuli at three different viewing distances, in both head-moving and head-stationary conditions. A stereo-viewing system provided ocular separation for stereo stimuli and monocular viewing of parallax stimuli. For each motion parallax stimulus, a point of subjective equality (PSE) was estimated for the amount of binocular disparity that generates the equivalent magnitude of perceived depth from motion parallax. Similar to previous results, perceived depth from motion parallax had significant foreshortening. Head-moving conditions produced even greater foreshortening due to the differences in the compensatory eye movement signal. An empirical version of the motion/pursuit law, termed the empirical motion/pursuit ratio, which models perceived depth magnitude from these stimulus parameters, is proposed.

## Introduction

The visual perception of depth is an important part of successful navigation and obstacle avoidance. While the human visual system can employ a variety of visual cues to object depth, the percept of depth created by the relative movements of objects in the scene is especially salient for the moving observer. This apparent relative movement of objectively stationary objects is created by the translation of the observer and is called motion parallax. Specifically, during the lateral translation we study, the observer's visual system maintains fixation on a particular stationary object in the scene by moving the eyes in the direction opposite the translation. Therefore, while the visual system ensures that this fixated object remains stationary on the observer's retina during the translation, presumably to maintain acuity for the visual information available at this location (Miles, 1998), the retinal image of objects nearer and farther than the fixation point move in opposite directions on the observer's retina. This combination of retinal motion and eye pursuit was noted as far back as the 1925 edition of von Helmholtz (1910/1925/1962, Vol. III, p. 371) where the passage concludes, “…the probability is that both of them generally contribute to (forming estimates of distance) in some way, although it would be hard to say exactly how.” We now understand geometrically how the ratio of these rates determines relative depth and experimentally why the motion/pursuit ratio is a key quantity.

**Figure 1:**
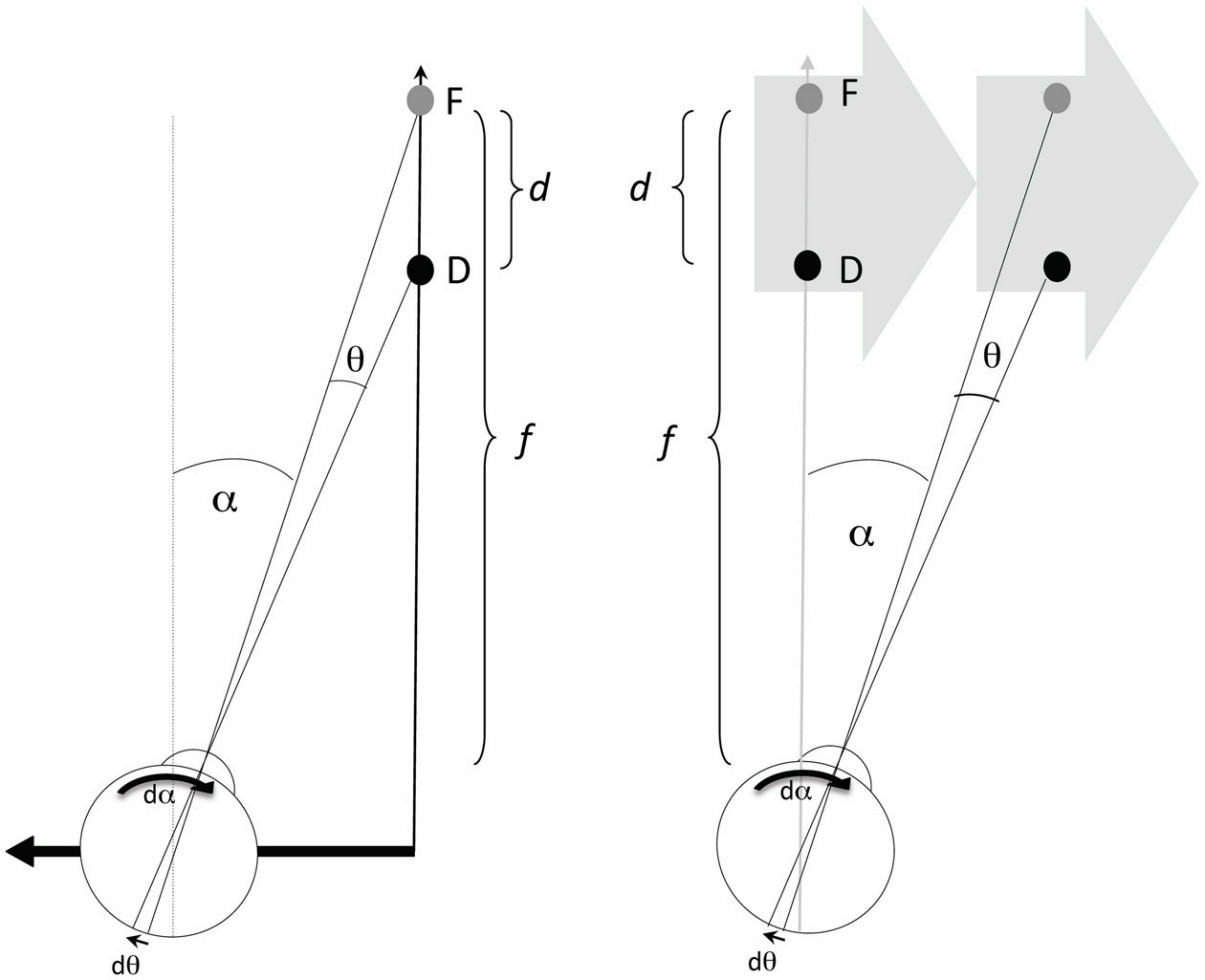
**The left panel depicts one condition producing motion parallax, with the eye (and head) translating laterally to the left**. Point F is the fixation point at viewing distance (*f*), and D is the point with some depth (*d*) beyond F. The value *d*α gives the increment of eye rotation necessary to maintain fixation on F during an increment of the translation. The value *d*θ/*dt* gives the velocity of the D on the retina. D in any other position would generate a different *d*θ increment with the same *d*α, and thus a different ratio. The right panel shows that the same values of *f, d*, *d*α, and *d*θ can be created with a translating stimulus and stationary observer.

Information about the direction and speed of both the retinal image motion and the pursuit eye movement are used by the visual system to recover the relative depth of objects in the scene (Nawrot, 2003; Naji and Freeman, 2004; Nawrot and Joyce, 2006; Nadler et al., 2009). The prototypical conditions for motion parallax (Figure 1, left panel) involve a translating observer maintaining fixation upon a static point (F) giving a viewing distance (*f*). The angle of the observer's eye (α) changes over time (at rate *d*α/*dt* or displacement *d*α in a small time increment), which corresponds to the magnitude of the observer's compensatory eye movement. While the fixation point remains stationary on the observer's retina, other points (illustrated here by point D) nearer or farther than the fixation point will move on the observer's retina by the change in angle θ (at rate *d*θ/*dt* or displacement *d*θ in a small time increment) which correspond to the magnitude of retinal image motion of D. The relationship between these values (*d*θ and *d*α) and relative depth (*d/f*), between points F and D, is geometrically given by the motion/pursuit law (M/PL) (1),

(1) $$\frac{d}{f}=\frac{\text{d}\theta }{\text{d}\alpha }\frac{\text{1}}{\text{1-d}\theta \text{/d}\alpha }$$

which describes how the visual system could use the retinal motion signal (*d*θ) and the eye movement signal (*d*α) to determine the exact ratio of depth (*d*) to viewing distance (*f*) (Nawrot and Stroyan, 2009; Stroyan and Nawrot, 2012). Because of the small value of the motion/pursuit ratio in our experiments, the exact geometric law (1) can be replaced with the simple approximate geometric relationship that says the motion/pursuit ratio (M/PR) approximates relative depth (2):

(2) $$\frac{d}{f}\approx \frac{\text{d}\theta }{\text{d}\alpha }$$

Of course, if the visual system has an available estimate of viewing distance (*f*), like the estimate of viewing distance required to recover depth from retinal disparity for binocular stereopsis, the M/PR could be used to describe the recovery of depth (*d*) from motion parallax in a process very similar to that for binocular stereopsis. In fact, there is even a strong geometrical similarity between the M/PR and the ratio of retinal disparity over binocular convergence (Stroyan, 2010). Further, there is some evidence that the brain may use “affine” quantities to represent quantities like depth (Di Luca et al., 2010), so the affine M/PR may even have a neural representation. Additional details of how the current “motion/pursuit ratio” approach differs from previous “observer velocity” approaches to motion parallax (e.g., Nakayama and Loomis, 1974; Longuet-Higgins and Prazdny, 1980) are provided in Nawrot and Stroyan (2009) and Stroyan and Nawrot (2012). Interactive numeric demonstrations of the motion/pursuit approach can be viewed at Stroyan (2008).

In addition to the case of a translating observer, the same M/PL describes the relationship when the observer is stationary (Figure 1, right panel) and viewing a translating stimulus (Graham et al., 1948). The primary difference between the two viewing conditions is that *d*α comprises a pursuit signal in the observer stationary case, while *d*α is a combination of pursuit and translational vestibular ocular response (tVOR) signals in the observer translation case. Previous work (Nawrot and Joyce, 2006) has shown that only the pursuit component of the *d*α signal is used for motion parallax. Therefore, these two conditions should produce different estimates of perceived depth magnitude. This is one of the hypotheses to be investigated here.

While the M/PR provides a reasonable approximation of the M/PL, neither provides an explanation for the perceptual underestimate of depth, or foreshortening, of perceived depth from motion parallax (e.g., Ono et al., 1986; Domini and Caudek, 2003; Nawrot, 2003). For instance, in two experiments Durgin et al. (1995) show motion parallax foreshortening between about 25% and 125% compared to comparable binocular disparity stimuli. This led them to conclude, “ … geometrically equivalent depth information does not lead to the same quantitative perception [of depth] when presented through motion parallax as when presented through binocular disparity.” This is clear evidence that motion parallax and binocular disparity generate different perceptual estimates of depth given the same underlying geometry in a scene.

More recently, McKee and Taylor (2010) reported that motion parallax difference thresholds are about 10 times larger than comparable thresholds with binocular disparity. While studying the precision of depth judgments in a “natural setting”—objects and rods presented on a stage—McKee and Taylor (2010) found that 8–10 cm lateral head translations did not improve static monocular depth thresholds for most observers at the 112 cm viewing distance. Moreover, depth thresholds for all three observers were about a log_10_ unit higher for motion parallax than to the comparable binocular disparity conditions. This indicates that observers exhibit much less sensitivity in the use of motion parallax, compared to binocular disparity, for the recovery of information about the geometry of a visual scene. The magnitude of the perceptual foreshortening suggested by Durgin et al. (1995) and by McKee and Taylor (2010) is large indeed, and presents a challenge to the purely geometric analysis provided by the M/PL and M/PR. Other important factors must be involved.

These other important factors are the accuracy of the actual eye movement and retinal image velocity signals recovered by the visual system. The depth estimate provided by the M/PR model assumes that the visual system has accurate internal signals regarding retinal image motion and the pursuit eye movement. While this is a reasonable starting point when considering the underlying geometry and how it might theoretically provide the information necessary to recover depth from motion parallax, this assumption of accurate motion signals is a less reasonable assumption for a model of human perception of depth from motion parallax. We know that the accuracy of perceived motion velocity is affected by disparate stimulus parameters such as contrast (Campbell and Maffei, 1981), color (Cavanagh et al., 1984), dot density (Watamaniuk et al., 1993), and spatial frequency (Diener et al., 1976; Campbell and Maffei, 1981). Moreover, the accuracy of internal eye movement signals, studied in the context of combination with retinal image motion for the perception of head-relative motion, can be quite inaccurate (Freeman and Banks, 1998; Freeman, 2001; Turano and Massof, 2001; Souman and Freeman, 2008). To model these inaccuracies, these studies have applied linear gain factors or non-linear transducers to the retinal image velocity and eye movement velocity signals to explain velocity-matching results. For instance, in one of the earliest explorations of how retinal image motion and pursuit eye movements affect perceived slant, Freeman and Fowler (2000) used a linear model of motion and pursuit combination to account for perceived-speed, which in turn explained changes in the perceived slant. We follow a similar rationale with the current study.

In the present work, the same retinal image velocity and eye movement velocity signals are employed, but for the markedly different purpose of recovering depth, not motion. It is interesting to know whether these signals display similar accuracy for the perception of depth from motion parallax as they do for the perception of head-centric motion. Therefore, the goal of the current study is to: (1) determine how well the M/PR predicts the perception of relative depth from motion parallax in psychophysical observers, and (2) determine whether non-linear transducers applied to the retinal motion signal and to the pursuit signal can produce an “empirical” M/PR model that accounts for the perception of depth magnitude from motion parallax.

## Materials and methods

Observers completed a 2IFC task comparing depth magnitude from a motion parallax stimulus with the depth from a binocular disparity stimulus (e.g., Nawrot, 2003; MacKenzie et al., 2008; Domini and Caudek, 2009, 2010). The experiment included six different conditions: two motion parallax with head-translating conditions at two viewing distances (36 and 72 cm), and four head-stationary conditions at three viewing distances (36, 54, and 72 cm). Two conditions were run at the 36 cm viewing distance with stationary head, each condition having a different range of pursuit (*d*α) speeds. Both conditions at 36 cm included the 4.95 d/s pursuit speed providing a partial replication of those data points. For each motion parallax stimulus, the point of subjective equality (PSE) between the two stimuli (*d_stereo_* ≈ *d*_*mp*_) allowed the particular stereo stimulus parameters to provide a reasonable estimate of the depth from a particular set of physical motion parallax parameters. It is then possible to compare empirical estimates of *d_mp_* to the theoretical depth predicted by the parameters of the M/PR, and determine how these empirical estimates differ from the geometric model.

The accuracy of the motion parallax depth magnitude estimates depends on how closely perceived depth from binocular disparity represents the binocular stimulus geometry (depth constancy). While there are examples of systematic distortions in perceived depth from binocular disparity (e.g., Johnston, 1991; Tittle et al., 1995; Todd and Norman, 2003), most failures of depth constancy are linked to a mis-estimate of viewing distance due to “reduced viewing conditions” (Wallach and Zuckerman, 1963; Cumming et al., 1991; Johnston et al., 1994; Durgin et al., 1995; Todd and Norman, 2003; Domini and Caudek, 2010). Therefore, the current study employs “full-cue” viewing conditions that optimize distance perception (Mon-Williams and Tresilian, 1999) and have lead to accurate depth perception (Philbeck and Loomis, 1997). Additionally, to optimize the inter-cue depth magnitude comparison, identical viewing conditions were used for both the motion parallax and binocular disparity stimuli. Any distortion in perceived depth resulting from a mis-estimate of viewing distance should affect both motion parallax and binocular disparity. Furthermore, in the Discussion Section below we have included an analysis of the data that uses a systematic error of the perception of stereoscopic depth based on Johnston (1991). The effect of that analysis is to determine the effect of such a systematic error in stereo depth constancy on the exponents of the empirical motion/pursuit ratio. This analysis shows the changes due to purported mis-estimate of depth from binocular stereopsis are small compared to the systematic under-estimate of perception of depth from motion parallax.

Here the “full-cue” conditions were implemented with a Z-Screen (Stereographics; San Rafael, CA) stereo viewing system that allows natural binocular viewing of the stimulus monitor without mirrors, prisms, or active shutter-glasses. Moreover, viewing distances were less than 80 cm, the distance within which convergence provides the most reliable cue to viewing distance (Von Hofsten, 1976; Ritter, 1977; Brenner and Van Damme, 1998; Brenner and Smeets, 2000; Mon-Williams et al., 2000; Viguier et al., 2001) and the distance within which accommodation may contribute as a cue to viewing distance (Fisher and Ciuffreda, 1988; Mon-Williams and Tresilian, 1999). These short viewing conditions, and the use of passive stereo-viewing glasses, ensured that the vertical disparity information available to scale the distance of the display and monitor (Garding et al., 1995; Rogers and Bradshaw, 1995; Bradshaw et al., 1996; Read and Cumming, 2006) was large and unobstructed. Therefore, these viewing conditions were optimized for the use of depth-scaling cues such as convergence, accommodation, vertical disparity, and their possible combination (Backus et al., 1999).

Moreover, the role of the particular psychophysical task has also been examined in the failure of depth constancy in binocular stereopsis (Frisby et al., 1996; Glennerster et al., 1996; Bradshaw et al., 1998, 2000; Todd and Norman, 2003). Psychophysical depth-matching tasks were found to produce more accurate depth constancy than shape-judgment tasks. Such depth-matching tasks are considered “Class A” observations (Brindley, 1970) in which the observer compares the two sensations of depth produced by viewing two stimuli. Class A observations are believed to be more direct than alternative Class B observations by avoiding their necessary mental transformations, as with, for example, a depth-to-half-height task, although haptic tasks have shown near perfect depth constancy, similar to visual tasks (Foster et al., 2011). For example, Glennerster et al. (1996), Bradshaw et al. (2000), and Todd and Norman (2003) used both Class A and B observations, and all found more accurate depth judgments with the Class A depth-matching task, with an average performance close to perfect constancy. Therefore, to improve the accuracy of depth perception, the current study employed a Class A depth-matching task in which observers compared the sensation of depth produced by viewing two similar stimuli.

While the full-cue conditions in the current experiment were intended to provide maximum information about viewing distance (*f*) and increase accuracy of perceived depth for both the binocular disparity and motion parallax stimuli, depth constancy with these binocular disparity stimuli was investigated in a separate control condition. This condition simulated the design of Glennerster et al. (1996; see also Bradshaw et al., 2000; Todd and Norman, 2003) to empirically determine whether the binocular disparity stimulus used here provided a reasonable estimate of perceived depth magnitude for the motion parallax stimuli. To foreshadow the results, the deviation from perfect depth constancy was very small, indicating that the use of this binocular disparity stimulus in a perceptual matching procedure was reasonable for the task of determining perceived depth magnitude from motion parallax.

### Apparatus

Stimuli were generated on a Macintosh computer (Apple; Cupertino, CA) and presented on an IIyama CRT (IIyama International; Oude Meer, The Netherlands) monitor (1600 × 1200 × 85 Hz). In head-movement conditions, head position was measured with a linear potentiometer (ETI Systems; Carlsbad, CA) using a head-movement recording device (described in detail in Nawrot and Joyce, 2006). Head position was registered in the computer at 85 Hz using a 16-bit multifunction I/O board (National Instruments; Austin, TX) connected to the head movement device. The device has excellent linearity (*r* > 0.999) and accuracy (<0.1 mm).

A Z-Screen (Stereographics; San Rafael, CA) stereoscopic imaging system, which uses reversing circular polarization for frame-sequential presentation of the stereo images, was used for all conditions of the experiment. While this system gave stereo separation for the stereo stimulus presentation, it was also used to restrict presentation of the motion parallax stimulus to the observer's right eye. That is, the motion parallax stimulus was visible only to the observer's right eye, while the fixation stimulus was visible to both the observer's right and left eye. This maintained the same vergence, accommodation, and vertical disparity information for both the motion parallax and binocular disparity stimuli. Transitions of the polarization state of the Z-Screen were controlled by the experimental computer through a digital output channel in the multi-function I/O board. With this stereoscopic viewing system, observers wore passive “aviator-style” glasses with the two lenses fitted with opposite directions of circular polarization, similar to the “Real3D” glasses commonly used in 3D movie viewing in theaters. The use of these glasses precluded the use of a remote-optics eye tracking system to verify observer fixation in this experiment. Previous work has compared conditions in which fixation was and was not objectively enforced with an eye tracker (Nawrot and Stroyan, 2009) and demonstrated very similar quantitative results in both conditions. Here, as in both conditions of Nawrot and Stroyan (2009), observers were given instructions about the importance of maintaining fixation.

To minimize any effect of cross-talk in the binocular viewing system (information presented to one eye that is visible to the other eye), the monitor luminance was reduced to 38.8 cd/m^2^, which was further reduced to 16.0 cd/m^2^ by the Z-screen viewing system. In a functional test of cross-talk, information was presented in one of the two channels to an observer with one eye occluded. Using the non-occluded and non-presented eye, observers were at chance in detecting whether or not a stimulus was presented and were at chance in detecting the direction of a translating stimulus.

In the depth-constancy control conditions, the viewing apparatus was duplicated with one monitor and Z-screen at a viewing distance of 36 cm (and offset to the left of the line of sight, similar to the virtual monitor positions in Figure 1 of Glennerster et al., 1996) and the other monitor and Z-screen at a viewing distance of 72 cm (and offset to the right of the line of sight). The height of the monitors was adjusted to make the centers of the two stimuli level with the observer's eye. Synchrony of the monitors was achieved by splitting the signals to both monitors and Z-screens. The stimulus viewed at 36 cm (left monitor) was drawn on the right side of the screen while the left side of the screen was occluded. The stimulus viewed at 72 cm (right monitor) was drawn on the left side of the screen while the right side of the screen was occluded.

### Stimuli

To allow comparison of these results to other studies in the motion parallax literature, we employed a random-dot stimulus depicting a frontal corrugated surface varying sinusoidally in depth along the vertical dimension (Rogers and Graham, 1979). The general design of this type of random-dot stimulus for stereo, head-movement, and head-stationary motion parallax conditions has been detailed elsewhere (Nawrot and Joyce, 2006). In the current experiment the stimulus depicted 1 cycle of depth corrugation, with one half-cycle appearing above and below the fixation point.

The square stimulus window was 300 × 300 pixels (0.244 mm/pixel). At the three different viewing distances this corresponded to: 11.5° (2.3 min/pixel) at 36 cm, 7.75° (1.55 min/pixel) at 54 cm, or 5.85° (1.17 min/pixel) a side at 72 cm viewing distance. The stimulus was composed of 5000 one-pixel black dots randomly positioned on a white background. The maximum disparity of the corrugated stereo stimulus varied between 1 and 9 pixels, with the angular dimension varying with viewing distance: 36 cm, 2.3–20.7 min; 54 cm, 1.55–14.0 min; 72 cm, 1.17–10.5 min. The horizontal meridian and the fixation point always had zero pixels of disparity. The stereo stimulus was stationary and drawn at the center of the monitor. Motion parallax stimuli varied between maximum *d*θ/*d*α ratios of 0.042 and 0.25 with a variety of pursuit (*d*α/*dt*, 1.1–11.57 d/s) and retinal image (*d*θ/*dt*, 0.14–1.65 d/s) velocities. Motion parallax stimuli were presented to the right eye, while the left eye was presented only the fixation spot. This allowed the fixation spot to be binocularly fused by the observer, ensuring the same ocular convergence and accommodation in both motion parallax and binocular disparity stimuli.

In head-stationary conditions, the motion parallax stimulus window translated 7.3 cm across the monitor at the specified *d*α velocity for that stimulus trial. Within the translating stimulus window, dots generating the peak motion parallax cue moved leftwards or rightwards at the peak *d*θ velocity for that trial. Since the observer maintained fixation on a point at the center of the translating stimulus window, these *d*θ stimulus velocities correspond to retinal image velocities. The duration of the stimulus presentation varied, and depended on the particular *d*α velocity.

In head-translation conditions, the motion parallax stimulus window remained stationary on the monitor and was only displayed during the central 7.3 cm of each trial's head translation. Observers were instructed to move at a speed so that the entire head translation took about 1 s, and the stimulus presentation duration was about 0.5 s. This corresponds to a commonly used 0.5 Hz head translation speed (e.g., Nawrot, 2003). The precise duration of the observer's head translation through the central 7.3 cm was recorded for each trial and was used to calculate the average *d*α and *d*θ values. The peak velocity of local stimulus dot movements (within the stimulus window) was linked to the velocity of the observer's head translation, which was measured every 0.012 s with the head movement device. Observers maintained fixation on a point at the center of the stimulus window with eye movements during the head translation, and local stimulus dots moved in relation to this point, making it possible to maintain the proper M/PRs (*d*θ/*d*α values between 0.042 and 0.25) for each trial, even though the exact head translation velocity varied between trials.

In the depth-constancy control conditions, two stereo stimuli were drawn to the screen at the same time. However, observers saw only the stimulus on the left side of the screen at the 72 cm distance, and the right side stimulus at the 36 cm distance. Stimulus dots viewed at 36 cm were 1 pixel (2.3 arc min) in size, and those viewed at 72 cm were 2 × 2 pixels (2.3 arc min) in size. In one condition the stimulus viewed at 36 cm was fixed at a peak disparity of 23.3 arc min, while variable stimulus at 72 cm varied between 1.2 and 11.7 arc min of disparity in a method of constant stimuli. In the second condition, the stimulus viewed at 72 cm was fixed at a peak disparity of 4.7 arc min of disparity while the variable stimulus at 36 cm varied between 9.3 and 28 arc min of disparity in a method of constant stimuli. Similar to the other conditions, the phase of the two stimuli was always reversed. Unlike other conditions, viewing of the two stimuli was unrestricted.

### Procedure

These procedures were overseen by the North Dakota State University Institutional Review Board and adhered to the tenets of the Declaration of Helsinki. Observers were required to have corrected acuity of 20/40, Pelli-Robson contrast sensitivity of 1.80, a stereothreshold (Randot and Stereofly tests) of 50 s, and not have neurological or ophthalmic disorders. Eight naïve observers performed a 2IFC of perceived depth magnitude between a motion parallax stimulus (first interval) and a binocular disparity stimulus (second interval). Trials began with a fixation dot positioned at the center position of where the motion parallax stimulus presentation would begin. Following the motion parallax stimulus presentation the fixation dot moved to the center of the display and following a 1 s ISI, the binocular disparity stimulus was displayed.

**Figure 2:**
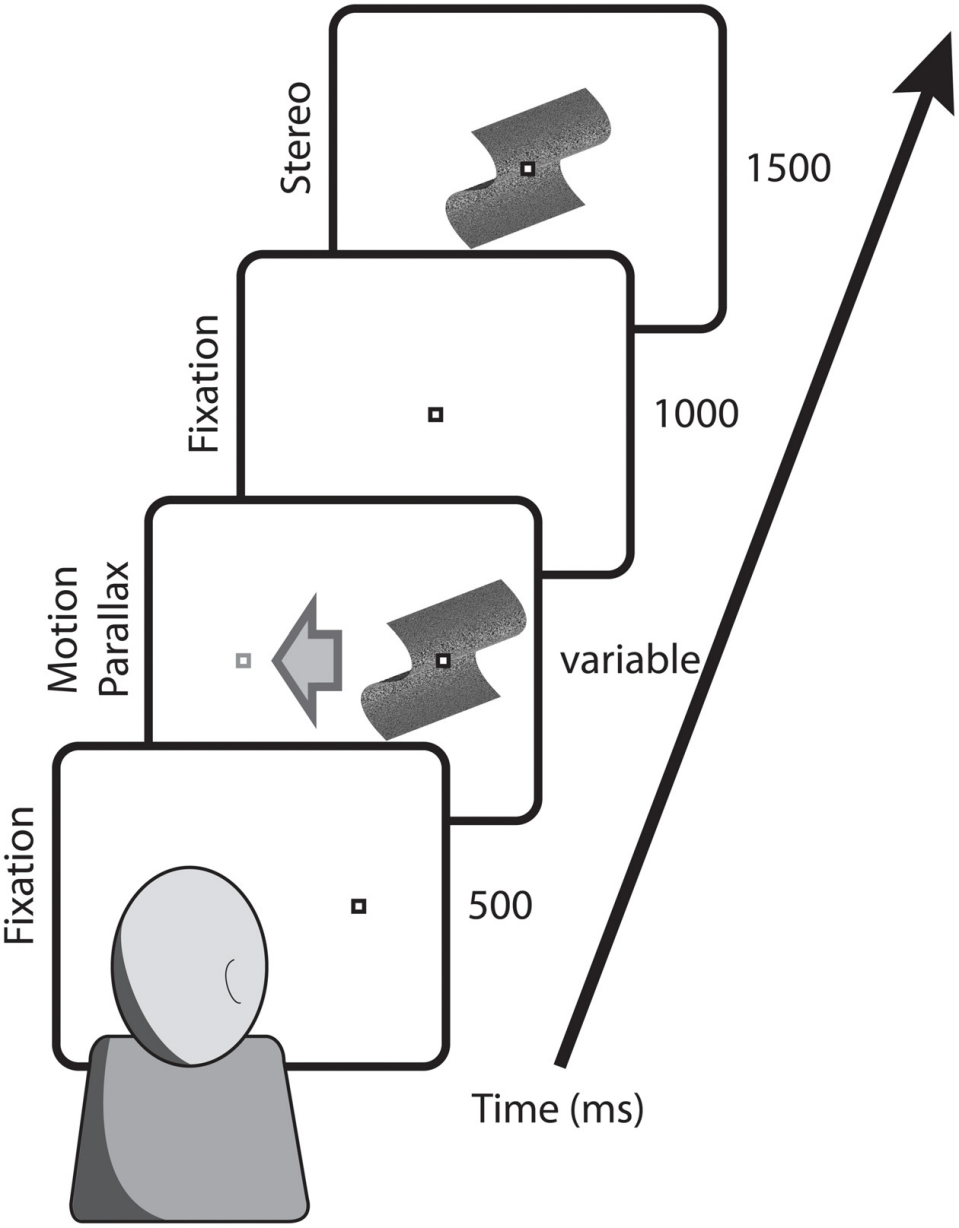
**Depicted are the key stimulus events in conditions with a stationary observer**. Trials began with the fixation point at the position that the motion parallax stimulus would appear and translate across the screen. Following a 1000 ms ISI, the comparison stimulus with binocular stereopsis stimulus appeared at the screen center.

**Figure 3:**
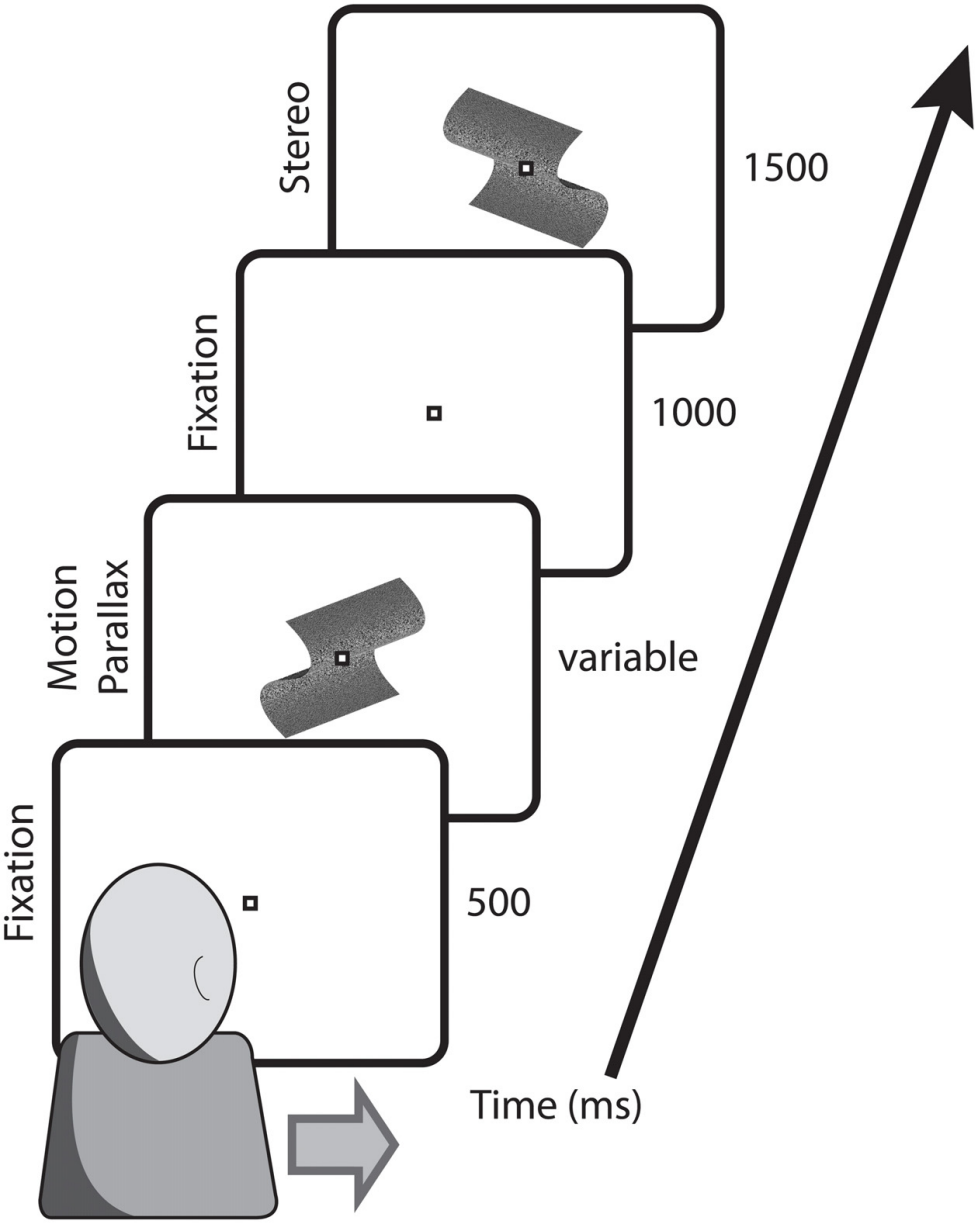
**Depicted are the key stimulus events in conditions with a translating observer**. Trials began with the fixation point at screen center and the observer's head extended to the side indicated on the screen. During observer head translation the motion parallax stimulus was presented at the screen center. Following a 1000 ms ISI, during which the observer's head was returned to a central position, the comparison stimulus with binocular stereopsis stimulus appeared at the screen center.

For head-stationary conditions (Figure 2), trials began with the fixation point displaced to the left or right of the monitor center, indicating the center of the motion parallax stimulus when the trial began. Observers initiated the trial with a button press. For head-translation conditions (Figure 3), a screen graphic indicated which direction (left or right) the observer was required to move his or her head during the trial. In both figures the stimuli are depicted with perspective information, but are actually perceived as fronto-parallel to the observer. To initiate the trial the observer was required to move his or her head to an appropriate starting position >5 cm from the center head position. When the observer's head was in an appropriate starting position, the graphic indicator vanished and the central fixation point appeared, indicating that the observer's head should then be translated across the display. The motion parallax stimulus was presented when the observer's head movement was within 3.65 cm of the center head position. The stimulus disappeared and the trial ended when the observer's head had traveled through the entire center 7.3 cm of head position. The stimulus disappeared and the trial was repeated if the observer's head movement stopped or reversed while within the central 7.3 cm range of head translation. During the 1 s ISI, the observer's head was moved to a central position and held stationary during presentation of the binocular disparity stimulus.

Following the presentation of the second stimulus the screen was blanked, and observers could then use a button press to indicate which of the two intervals contained the stimulus with the larger magnitude depth. Following the response, the appropriate fixation point was drawn to the screen indicating the observer could initiate the next trial. Each of the eight observers completed 20 blocks of 117 trials in each of the 6 conditions (~14000 trials). Leftward and rightward directions of head and eye movements alternated, and the two directions were collapsed in the subsequent analysis.

The experiment included six different conditions: two motion parallax with head-translating conditions at two viewing distances (36 and 72 cm), and four head-stationary conditions at three viewing distances (36, 54, and 72 cm). Two conditions were run at the 36 cm viewing distance with stationary head, each condition having a different range of pursuit (*d*α) speeds. Both conditions at 36 cm included the 4.95 d/s pursuit speed providing a partial replication of those data points.

In the depth-constancy control conditions, trials began with two fixation spots drawn where the two stationary stereo stimuli would appear. Nine naïve observers completed two blocks of 90 trials in two separate conditions. In each of the two control conditions, the peak stimulus disparity at one distance was held constant while the peak disparity at the other distance was varied. Observers initiated each trial with a button press. Both stereo stimuli were presented simultaneously and observers were free to move their gaze back and forth to compare the two stimuli. Observers used a button press to indicate which of the two stimuli appeared to have greater depth. Following the response both stimuli were extinguished, and the fixation spots were redrawn signaling the start of the next trial.

## Results

### Control conditions

**Figure 4:**
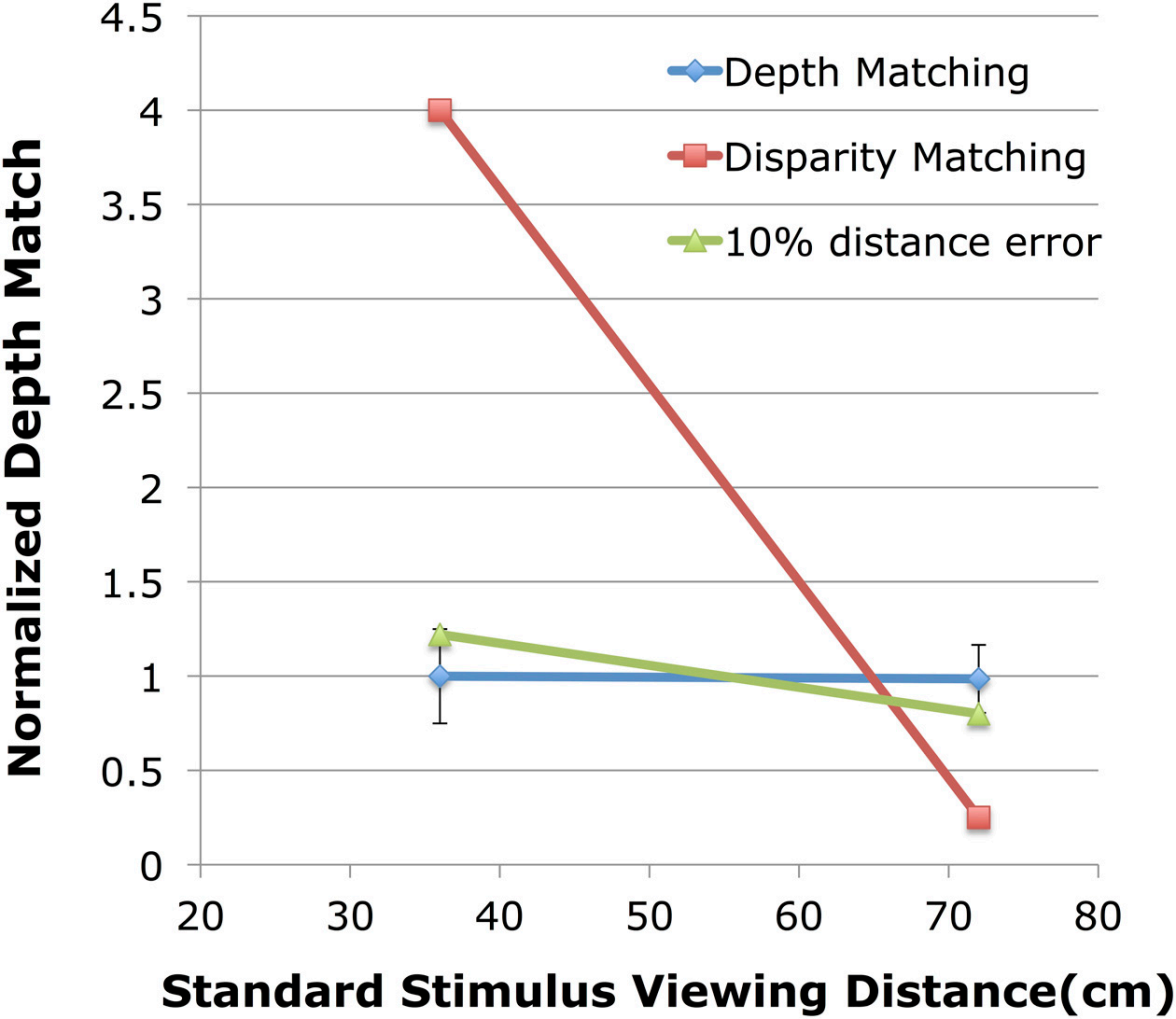
**Shown are the normalized depth matches for the control experiment compared to two hypothetical results**. The blue line shows the normalized depth match (match/expected) on the vertical axis with the viewing distance of the fixed standard stimulus shown on the horizontal axis. The red line shows the expected results if observers were matching disparity. The green line shows the expected results if the viewing distance to the variable stimulus were mis-estimated by 10%.

For each observer, in each of the two control conditions, a PSE was determined from each psychometric function based on a cumulative normal. This PSE gives the binocular disparity of the variable stereo stimulus at one depth that appears to match the magnitude of depth from the fixed binocular disparity of the stereo stimulus viewed at the other distance. Figure 4 shows the normalized depth matches found in the control condition. The blue symbols show the results of the two control conditions. The red symbols give the hypothetical results if observers were matching retinal disparity of the two stimuli instead of relative depth. The green symbols give the hypothetical results of the depth matching if observers had a 10% mis-estimate of viewing distance to the variable stimulus, an overestimate when the variable stimulus was at 36 cm, and an underestimate when the variable stimulus was at 72 cm.

When the standard stimulus, viewed at 36 cm (Figure 4, left blue point), was fixed at 23.3 arc min of peak disparity, the average matching stimulus at 72 cm viewing distance had 5.82 (*SE* = 0.16) arc min of disparity. In terms of depth, the fixed stimulus at 36 cm had 1.35 cm of depth while the variable stimulus at 72 cm was judged equivalent when it had 1.349 (*SE* = 0.04) cm of depth for a normalized depth match of 0.999. Similarly, a psychometric function fit to the cumulative data produced a PSE estimate of 5.80 arc min with β = 0.656 arc min, and σ = 1.08 arc min. This corresponds to a depth discrimination threshold of 0.25 cm for the binocular disparity stimulus, and corresponds to the left error bar shown in Figure 4.

When the standard stimulus, viewed at 72 cm (Figure 4, right blue point), was fixed at 4.66 arc min of peak disparity, the average matching stimulus at 36 cm viewing distance had 18.37 (*SE* = 0.41) arc min of disparity. In terms of depth, the fixed stimulus at 72 cm had 1.08 cm of depth while the variable stimulus at 36 cm was judged equivalent when it had 1.065 (0.03) cm of depth for a normalized depth match of 0.985. The psychometric function fit to the cumulative data produced a PSE estimate of 18.36 arc min with β = 0.228 arc min, and σ = 3.11 arc min. This corresponds to a depth discrimination threshold of 0.18 cm for the binocular disparity stimulus and corresponds to the right error bar shown in Figure 4. In both conditions observers were very accurate in their ability to match depths across a doubling of viewing distance. This depth constancy is not unexpected (see Materials and Methods). Indeed, the performance here is very similar to the performance of observers in Glennerster et al. (1996, see their Figure 2A).

These results indicate near perfect depth constancy for the binocular disparity stimuli viewed at the range of distances, and in the particular viewing conditions, used in this study. Such matches would only be possible if depth from each of the two binocular disparity stimuli were accurately scaled with their respective viewing distances. While Glennerster et al. (1996) point out that these results do not preclude a systematic mis-estimation of viewing distance (f), it is crucial that any mis-estimation preserved the precise viewing distance ratio used here. This alternative explanation appears unlikely for several reasons: First, the failure of depth constancy (e.g., Johnston, 1991) has often been attributed to a mis-perception of viewing distance that varied with the viewing distance (Johnston et al., 1994), being over-estimated at near distances and under-estimated at far viewing distances. Such a viewing distance-dependent pattern of mis-estimation is unlikely to preserve a precise ratio of viewing distances required for accurate depth constancy. That is, if the viewing distances were misestimated, the closer would be over estimated and the farther underestimated, disrupting the precise ratio necessary for this alternative explanation for depth constancy. Second, the purposeful discrimination of distance ratios, as required here, does not appear to be accurate enough (~5% error within 1 m, Baird and Biersdorf, 1967) to provide an alternative explanation for the accurate depth constancy. Moreover, the error in determining viewing distance ratios was even larger over longer viewing distances (see Table 4 in Baird and Biersdorf, 1967) such as those used in Glennerster et al. (1996) and Bradshaw et al. (2000) making the distance-ratio matching hypothetical a less likely explanation in those cases. Finally, there is no evidence that observers can actually attempt to match the ratio of two retinal disparites to the inverse ratio of the two viewing distances squared. In this control experiment, observers were asked to indicate which of the two stimuli appeared to have greater peak-to-trough depth, a task that they reported was very easy to complete (similar to the reports in Glennerster et al., 1996; Todd and Norman, 2003). One might reasonably conclude that depth matching is likely the product of a direct, low-level visual function relying on disparity sensitivity (Barlow et al., 1967) and low-level scaling by viewing distance (Trotter et al., 1996; Dobbins et al., 1998; Gonzalez and Perez, 1998), and there is no requirement that it be supplanted by an indirect, high-level, hypothetical distance-ratio computation. Therefore, we contend the current depth-matching data represents accurate depth constancy in these viewing conditions indicating that the binocular disparity stimuli used in the main experiment provide a reasonable means to estimate perceived depth from motion parallax.

### Experimental conditions

For each observer, in each condition, the 20 blocks of trials were compiled and used to generate a series of psychometric functions, one for each motion parallax M/PR value. Each psychometric function shows the percentage of judgments of the binocular disparity stimulus having greater depth than the motion parallax stimulus, for the nine different disparity values. The 50% PSE for each function gives the magnitude of binocular disparity (δ) that produced a perceived depth magnitude (*d_stereo_*) equivalent to the perceived depth magnitude for the motion parallax stimulus (*d_mp_* = *d*_*stereo*_) with the particular values of *d*θ, *d*α, and *f*. Knowing the binocular stimulus viewing parameters *d, f* and inter-ocular distance (*i*), it is possible to estimate *d_stereo_* from the distance-square law (3), and therefore recover a reasonable estimate of *d_mp_*.

(3) $$d_{mp}\approx d_{stereo}\approx \frac{f^{2}*\delta }{i}$$

**Figure 5:**
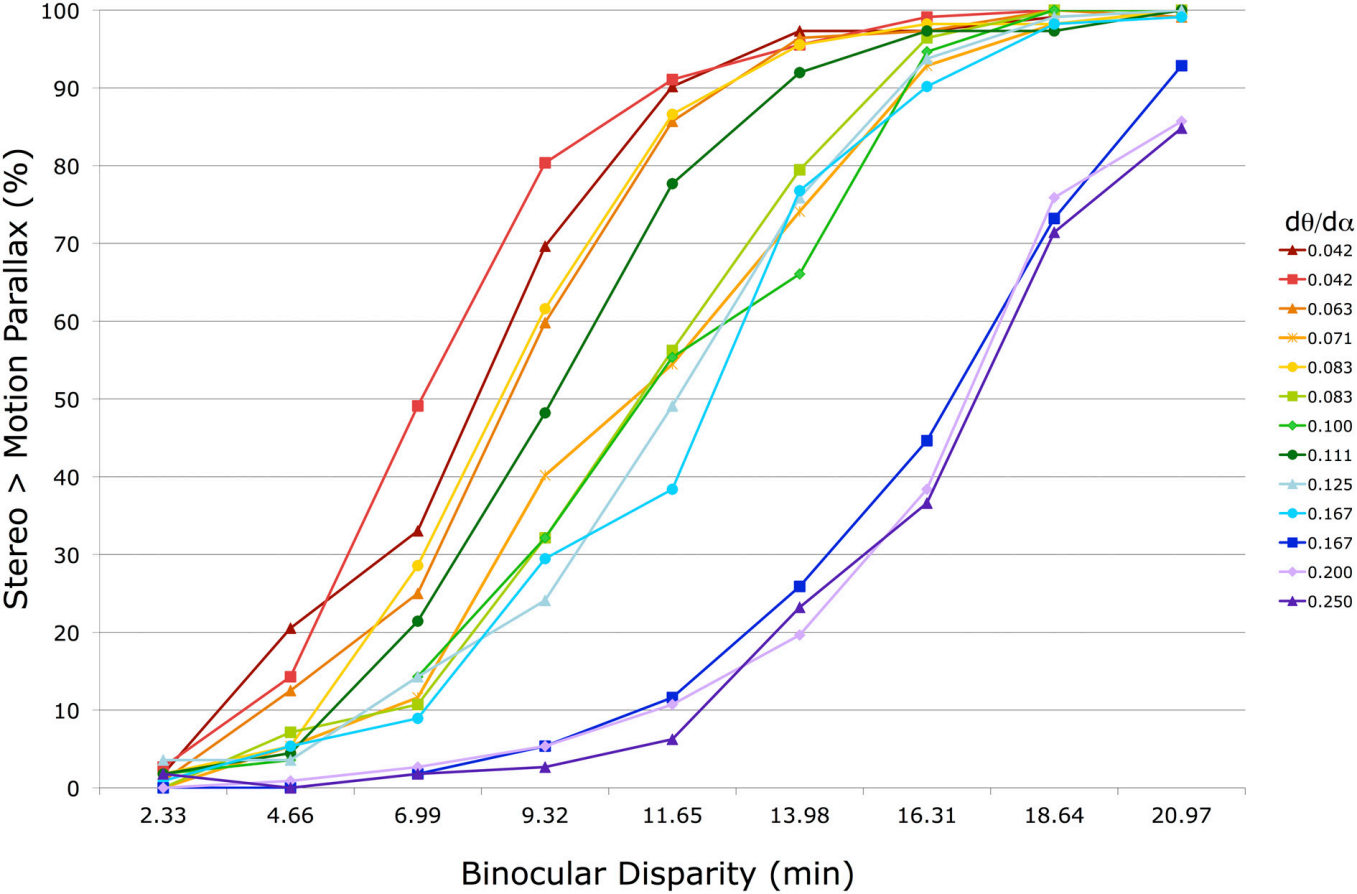
**Shown are 13 psychometric functions for group-averaged data in one head stationary condition with a 36 cm viewing distance**. The horizontal axis shows the peak disparity of the comparison binocular stereopsis stimulus. The vertical axis shows the percentage of trials in which the comparison stimulus was indicated to have greater depth magnitude than the motion parallax stimulus. The 13 different functions represent motion parallax stimuli with different motion/pursuit ratios (see legend). Lines with the same ratio are produced with different *dθ* and *d*α velocities.

Figure 5 shows the 13 raw psychometric functions for the group-averaged raw data in one condition (*f* = 36 cm, head stationary). Each line corresponds to a motion parallax stimulus with a different M/PR (*d*θ/*d*α) (see legend on the right). In a few instances (0.042, 0.083, and 0.167) the same M/PR is produced with different *d*θ and *d*α values. The horizontal axis shows the binocular disparity of the stereo stimulus being compared to the motion parallax stimulus. The vertical axis shows the percentage of responses for which the perceived depth magnitude of the stereo stimulus was greater than for the motion parallax stimulus. To the left side of the figure, with small disparities, the stereo stimulus is rarely perceived as having greater depth. To the right side, with large disparities, the stereo stimulus is most often perceived as having greater depth.

The psychometric functions, and PSE's, of seven observers were very similar in all 6 conditions. The remaining observer generated PSE's that were >3 SD from the group means, and were excluded from the subsequent group analysis. For each individual and group-averaged psychometric function, in each of the 6 conditions, the *d_mp_* was determined from the PSE of a fitted cumulative normal (ERF) in MATLAB (Mathworks; Natick, MA). For instance, 13 of these PSE's were determined from the data shown in Figure 5. The *d_mp_* estimates determined from group-averaged data were the same as the average of the individual *d_mp_* estimates. Standard error for the average *d_mp_* was calculated from the variability of these individual *d_mp_* estimates.

**Figure 6:**
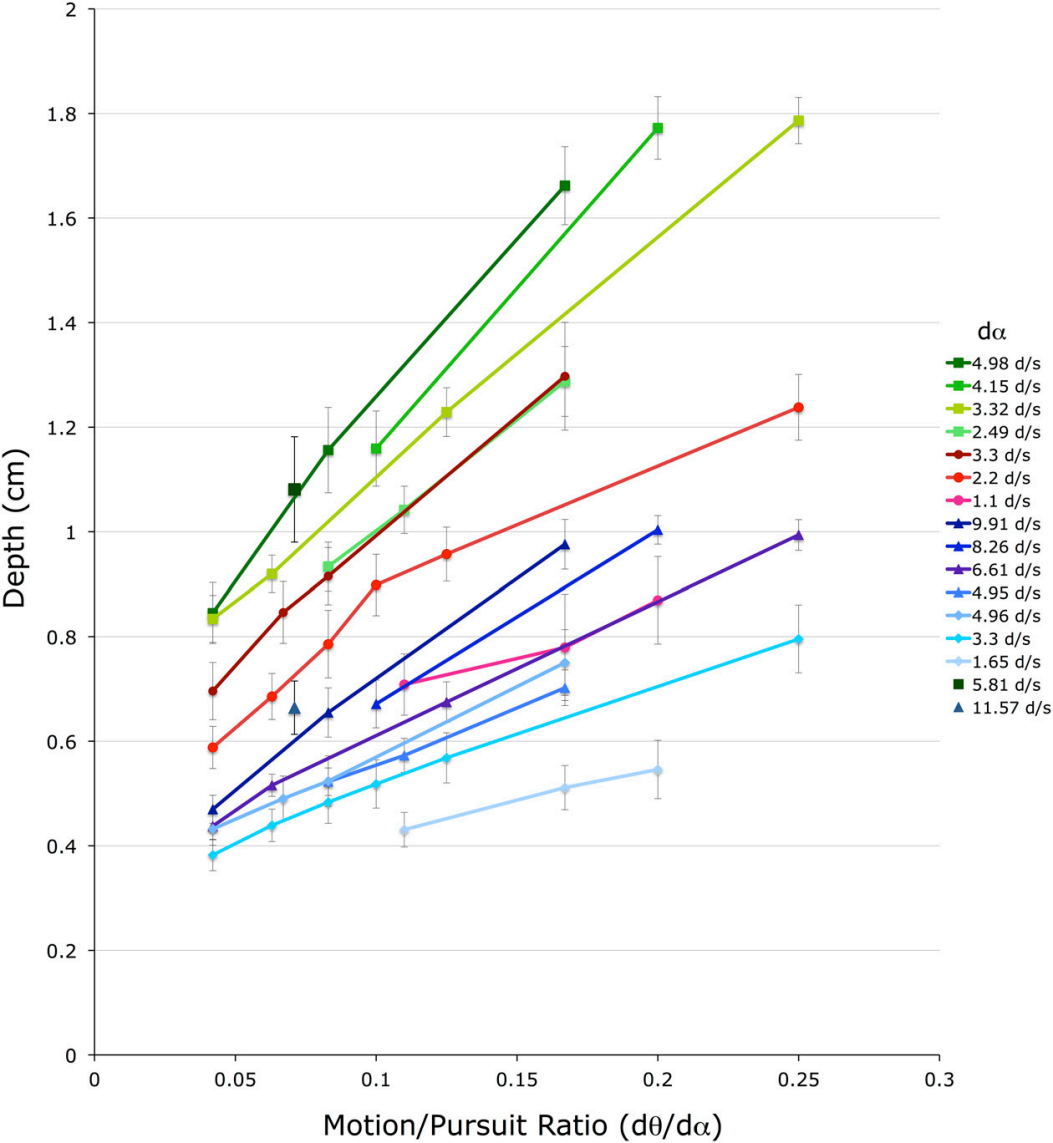
**Shown are the average depth matches in the four head stationary conditions**. The vertical axis shows depth depicted in the matching binocular disparity stimuli, the horizontal axis shows the different motion/pursuit ratios for motion parallax stimuli. Lines connect stimuli with the same pursuit velocity (*d*α, see legend) from the same condition. Lines and symbols shaded in blue are from conditions with 36 cm viewing distance, lines and symbols shaded in red are from 54 cm viewing distance, and lines and symbols shaded in green are from 72 cm distance. Two lone data points (5.81 d/s @ 72 cm, and 11.57 d/s @ 36 cm) are unaccompanied by other data points at that pursuit velocity.

Figure 6 shows these 52 *d_mp_* values for the 4 conditions with a stationary head. The horizontal axis shows M/PR (*d*θ/*d*α). The vertical axis shows depth depicted in the matching binocular disparity stimuli, providing an estimate of *d_mp_*, in cm. The different color groups correspond to the three different viewing distances (*f*) (greens = 72 cm, reds = 54 cm, blues = 36 cm). Lines connect *d_mp_* values that come from stimuli that have the same pursuit velocity (*d*α) (see legend) at the same viewing distance.

Several observations and conclusions can be made from this data. First, the magnitude of *d_mp_* is much less than that predicted from the geometric M/PR model. For instance, a M/PR of 0.25 and a viewing distance (*f*) of 36 cm should produce a *d_mp_* of 9 cm, but the largest *d_mp_* found in these conditions was about 1 cm. The *d_mp_* estimates for the 54 cm and 72 cm viewing distances were similarly an order of magnitude less than that predicted by the geometric model. A subsequent analysis will quantify this pattern of foreshortening for all of the stimulus variables.

Despite the foreshortening, *d_mp_* is still very orderly and varies with the *f*, *d*θ, and *d*α variables. Illustrating this orderly relationship, data from the three viewing distances shows an orderly increase in *d_mp_* with an increased in viewing distance (*f*) (Ono et al., 1986). In Figure 6 viewing distance is color coded with green points corresponding to 72 cm, red points to 54 cm, and blue points to 36 cm. The three colors are dispersed vertically meaning that points with similar *d*α and M/PR values produce different *d_mp_* values depending on the viewing distance. This distance scaling is predicted by the M/PR geometry, and appears very orderly with the points for the 54 cm viewing distance falling between those for the 72 cm and 36 cm viewing distances.

Additionally, data points form straight lines along each *d*α parameter, with each line sloping upward indicating a linear increase in *d_mp_* with the increase in the M/PR. This change in M/PR is accomplished here with a change in the *d*θ value, since *d*α is constant along each line. This shows the well-known role of *d*θ in the perception of depth from motion parallax. That is, with other independent variables remaining constant (*d*α and *f*), an increase in retinal image velocity (*d*θ) produces an increase in *d_mp_*. The direction and linearity of the *d*θ effect is predicted by the M/PR, but, as outlined above, the quantitative changes are less than that predicted by the geometric model.

**Figure 7:**
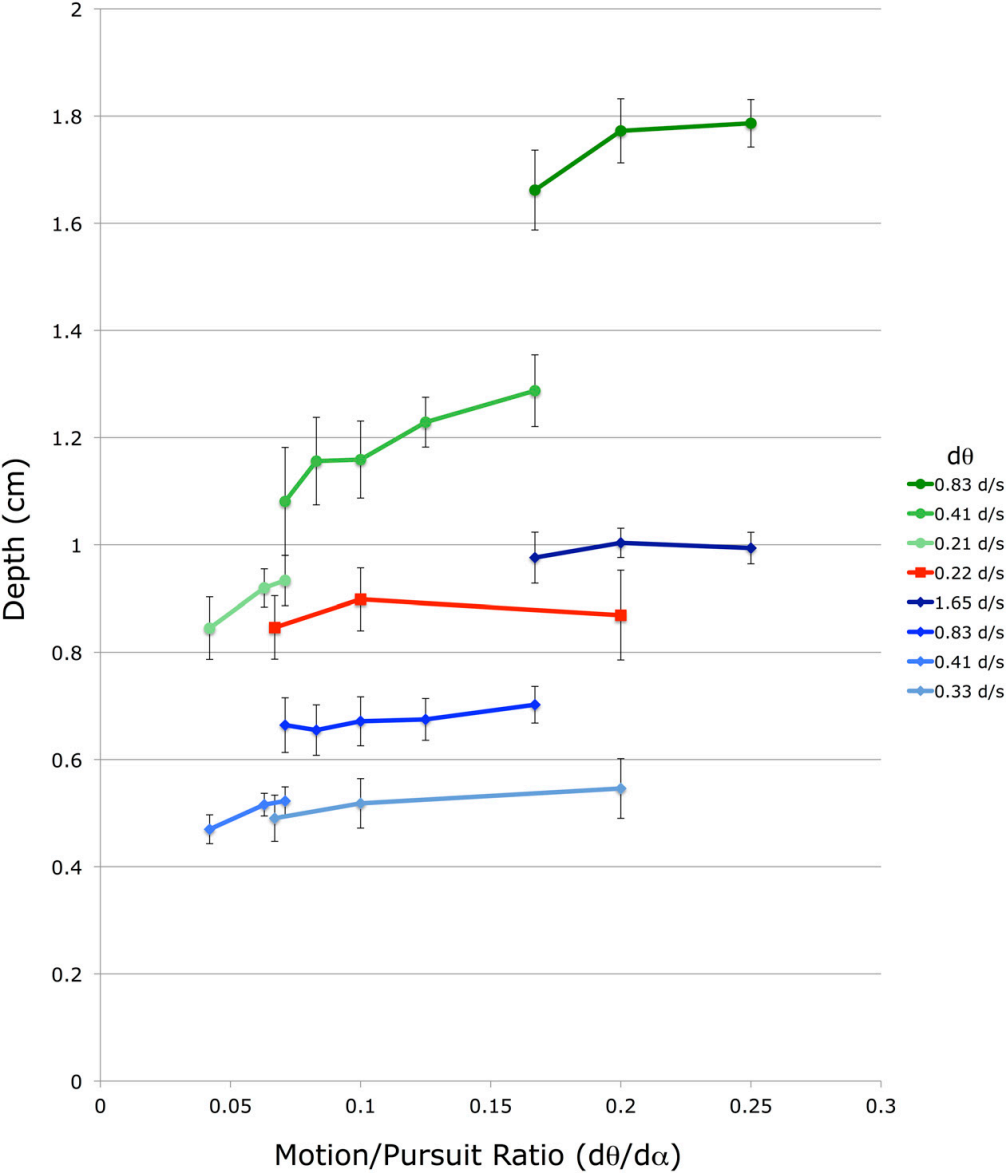
**Shown is a subset of the average depth match data from Figure 5**. The vertical axis shows depth depicted in the matching binocular disparity stimuli, the horizontal axis shows the different motion/pursuit ratios for motion parallax stimuli. Here lines connect stimuli with the same retinal image velocity (*d*θ, see legend) from the same condition. Lines and symbols shaded in blue are from conditions with 36 cm viewing distance, lines and symbols shaded in red are from 54 cm viewing distance, and lines and symbols shaded in green are from 72 cm distance.

A similar, but smaller, effect is found for changes in *d*α. The different lines in Figure 6 represent data points with different *d*α values, and within a particular viewing distance lines with smaller *d*α values produce smaller *d_mp_* magnitudes than lines with larger *d*α values. However, the vertical displacement of these *d*α lines is due to a change in both *d*α and *d*θ, as the M/PR remains constant. The independent effect of *d*α is most easily seen in Figure 7, which re-plots a subset of the points from Figure 6 for which at least 3 points share a common *d*θ value in the same viewing condition. The axes and data points are the same as Figure 6 but the lines now connect a fixed *d*θ value. Like the *d*α lines in Figure 6, these *d*θ lines also slope upwards with increasing M/PR (and therefore increasing *d*α), but with shallower slopes.

**Figure 8:**
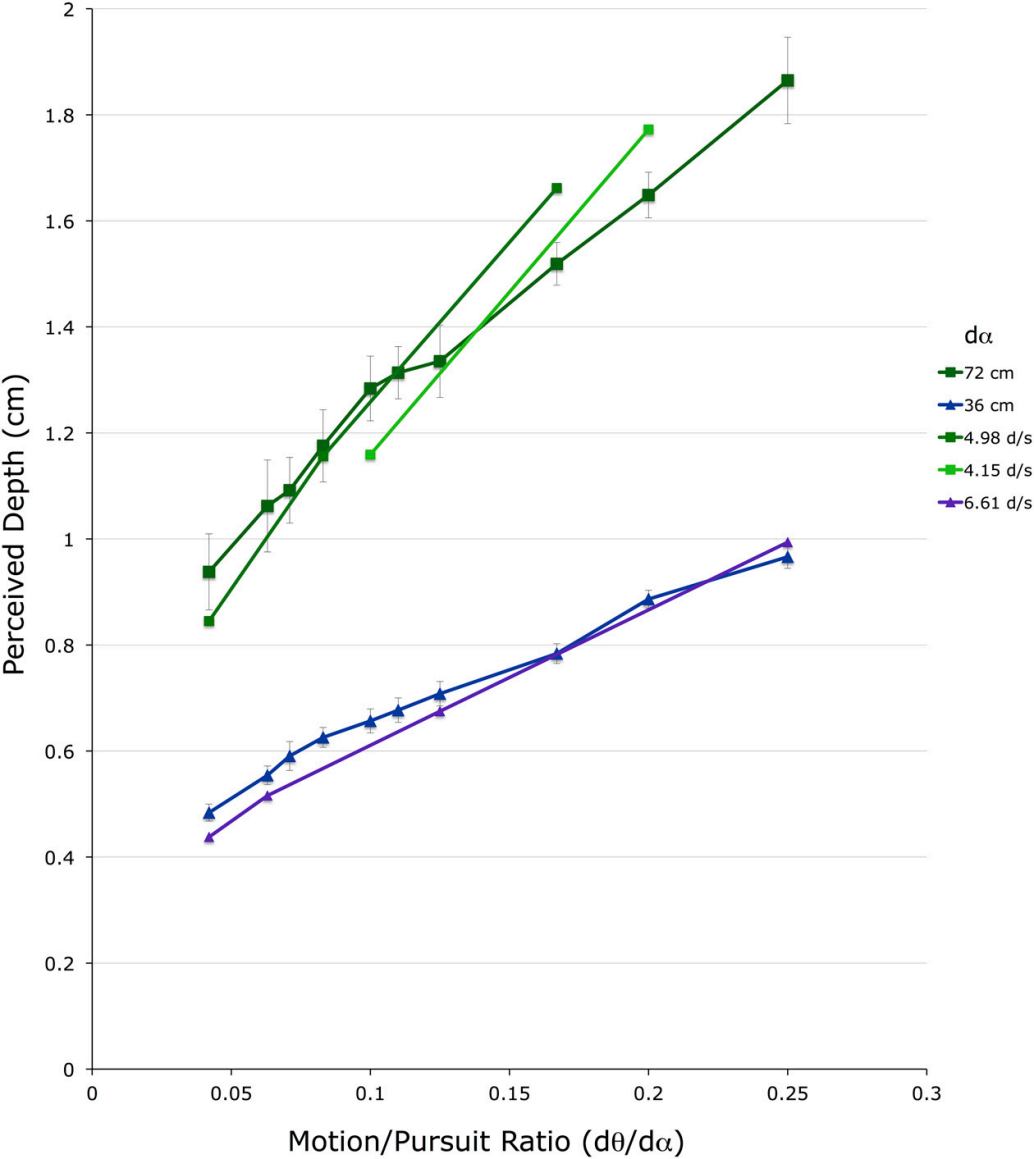
**Shown are the average depth matches in the two head translating conditions**. The vertical axis shows depth depicted in the matching binocular disparity stimuli, the horizontal axis shows the different motion/pursuit ratios for motion parallax stimuli. Lines connect stimuli with the same pursuit velocity (*d*α) from the same condition. The line and symbols shaded in blue are from the 36 cm viewing distance condition while those shaded in green are from the 72 cm distance condition.

Figure 8 shows 20 *d_mp_* values for the 2 conditions in which observers made lateral head translations. For comparison, the closest data points from the head-stationary conditions in Figure 5 are shown overlaid, without error bars. Again, the vertical axis shows depth depicted in the matching binocular disparity stimuli, providing an estimate of *d_mp_*, in cm The horizontal axis shows the M/PR (*d*θ/*d*α). The different colored points correspond to the two different viewing distances (*f*) (green = 72 cm, blue = 36 cm).

To determine the actual head translation speed, and the actual eye movement speed, an average head velocity was determined for each trial, for each observer, from the mean head velocity during the central 7.3 cm range of head translation. The mean observer head translation speed in the 72 cm viewing distance condition was 12.1 cm/s (*SE* = 1.2 cm/s), and in the 36 cm viewing distance condition was 11.0 cm/s (*SE* = 0.9 cm/s). With the assumption that the observer maintained accurate fixation on the static fixation point during the stimulus presentation and head translation, these head translation velocities correspond to an average eye movement speed (*d*α) of 9.3 and 17.5 d/s, respectively. It is important to note that regardless of the variability in the observer head translation speeds, the stimulus presentation program maintained the proper M/PR for each trial. However, knowing the average eye movement speed allows these results to be compared to those for the head-stationary conditions.

In the comparison of the 36 cm conditions, the blue line (with error bars) showing data from the head-translating condition shown in Figure 8 straddles the 6.6 d/s line (violet line) from the head-stationary condition shown in Figure 6. This similarity in the perceived depth suggests that at the same M/PR, a *d*α of 17.5 d/s during head translation produces the same *d_mp_* magnitude as a *d*α of 6.6 d/s in head-stationary conditions. In the comparison of the 72 cm conditions, the dark green line (with error bars), showing data from the head-translating condition shown in Figure 8, straddles the 4.98 d/s line (medium green) and the 4.15 d/s line (light green) from the head-stationary condition shown in Figure 6. Again, a comparison of the head stationary and the head moving conditions indicates that at the same M/PR, a *d*α of 9.3 d/s during head translation produces the same magnitude of *d_mp_* as *d*α of 4.15–4.98 d/s in head-stationary conditions.

The difference in the type of eye movements generated in the two conditions may explain this discrepancy: lateral head translations generate a tVOR in addition to the visually driven pursuit eye movement (Miles and Busettini, 1992; Miles, 1993). However, only the pursuit component of the compensatory eye movement is used in the perception of depth from motion parallax (Nawrot, 2003; Nawrot and Joyce, 2006). The tVOR signal does not appear to have role in the mechanisms serving perceived depth. Therefore, the internal *d*α signal generated during lateral head movements may be much less than the magnitude of the total compensatory eye movement generated during the lateral head translation. This was the rationale offered by Nawrot and Joyce (2006) to explain the transition and reversal in perceived depth sign between world-fixed and head-fixed motion parallax stimuli.

It appears that across a variety of viewing conditions, tVOR generates about 60% of the eye-movement compensation necessary to maintain fixation (Ramat and Zee, 2003; Liao et al., 2008). This means that to maintain fixation, and high visual acuity, the remaining 40% of the compensatory eye movement must come from a visually driven pursuit signal (Miles and Busettini, 1992; Miles, 1993). In the current experiment, we determined the eye movement velocities for which a head-stationary pursuit signal (36 cm: 6.6 d/s; 72 cm: 4.15 d/s) generates the same *d_mp_* magnitude as a head-translating tVOR+pursuit signal (36 cm: 17.5 d/s; 72 cm: 9.3 d/s). These pursuit velocities are about 40% (36 cm: 38%; 72 cm: 45%) of the tVOR+pursuit velocity. Therefore, the differences in perceived depth in the head-stationary and head-translating condition are explained by the differences in the eye movements, and support the proposal that the *d*α signal comes solely from the pursuit system (Nawrot and Joyce, 2006; Nadler et al., 2009).

## Discussion

The results indicate that depth from motion parallax is greatly foreshortened compared to the depth that might be expected from the dynamic geometry. Here, foreshortening means the object is perceived closer to the point of fixation than the spatial geometry indicates. For objects farther than the fixation point foreshortening means they are perceived nearer to the observer. But for objects closer to the observer than the fixation point, foreshortening means they are perceived as farther away from the observer than they actually are. Even with binocular, full-cue conditions that should provide a reliable estimate of physical viewing distance, (which might otherwise affect depth scaling) the depth foreshortening found here represents a near 10-fold diminution of perceived depth magnitude, which is further explained in the analysis below.

**Figure 9:**
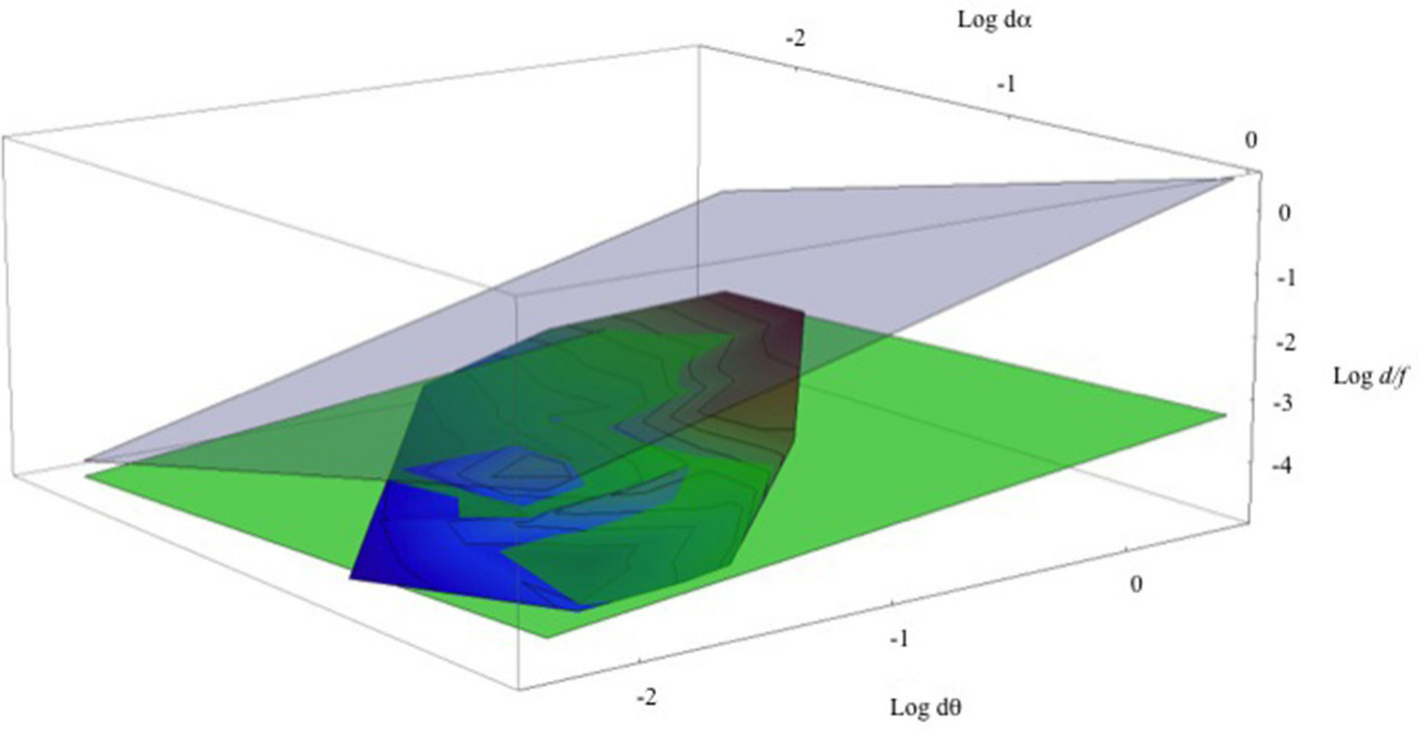
**Shown is a Log-Log-Log plot of relative depth (Log (*d*/*f*)) on the vertical axis, retinal image velocity (Log *dθ*) on the horizontal axis, and pursuit velocity (Log *dα*) on the (upper) z-axis**. The rainbow-shaded surface contains all of the data points from Figure 5. The green-shaded surface represents the least-squares fit to these data points. The gray-shaded surface represents veridical depth from the motion/pursuit law.

Returning to head-stationary conditions and the set of data points shown in Figure 6. Figure 9 shows a three-dimensional contour plot of this same data using *Log* (*d*α), *Log* (*d*θ), and *Log* (*d/f*). (Note that these are natural Logs, not Log_10_, and taking logarithms makes the M/PR (2) an exactly planar graph, $Log\left(\frac{d\theta }{d\alpha }\right)=Log\left(d\theta \right)-Log(d\alpha )$). Here the aggregate data from the three different viewing distances defines a remarkably flat contour shown with the rainbow coloring. The contour lines show equal relative depth (*d/f*). The overlain green plane depicts the least-squares fit to the data set (*Log* (*d/f*) = −3.463 + 0.416 *Log* (*d*θ) − 0.192 *Log* (*d*α). This agreement between the green plane and the data is excellent, with the *r*^2^ = 0.875. (A dynamic, rotatable version of this graph, and the program and the data points used to generate it, can be found in a Mathematica CDF file in the Supplementary Material). Of course, this least-squares fit does not represent a test of the relationship between the variables of the M/PR model but instead it provides a quantitative estimate of the relationship between the variables for the M/PR to explain the perceived depth measured here (e.g., Tufte, 1974/2006). The gray transparent plane illustrates the geometrically correct depth percept predicted by the MP/R. As noted earlier, perceived depth from motion parallax is greatly foreshortened compared to the depth predicted by the geometric model.

One possible reason is that the visual system is unable to recover, or to use, accurate motion or pursuit signals. The M/PL models a precise depth percept based on having veridical signals regarding *d*θ, *d*α, and *f*. The perception of motion during eye movements is an important problem in visual science (Mack and Herman, 1972; Brenner and van den Berg, 1994; Turano and Heidenreich, 1996). Incorrect estimates of the two dynamic signals, *d*θ or *d*α, could produce a misestimate of perceived depth magnitude (*d*_*mp*_), but the perceived underestimate seems to involve more than just estimates of the basic rates. The issue is how the visual system represents and then combines internal signals about retinal image motion and eye movement to generate an internal representation object movements in a scene. And, the visual system's solution to this problem is often inaccurate, as seen with the Aubert-Fleischl phenomenon (Fleischl, 1882; Wertheim and Van Gelder, 1990), in which the visual pursuit of an object reduces its perceived speed, and the Filehne Illusion (Filehne, 1922) in which a stationary object appears to move in the direction opposite an eye movement. One approach to this problem is to understand the inherent errors in the internal eye movement and retinal motion signals (e.g., Freeman and Banks, 1998) and model these errors with power-law transducers (Freeman, 2001; Turano and Massof, 2001; Souman and Freeman, 2008). These transducers give the estimated internal eye velocity and retinal image velocity signals based on the actual physical velocities.

The equation for the least-squares surface (the green surface in Figure 9) in non-log form gives the empirical motion/pursuit ratio:

(4) $$d_{mp}=\frac{\text{d}\theta^{\text{0}.\text{416}}}{d\alpha^{0.192}}\left(0.0313\right)f$$

This gives a result similar to Freeman (2001) and Turano and Massof (2001), where the log-least-squares coefficients act like power-law transducers (e and r) for the pursuit velocity signal (*d*α^e^) and for the retinal image velocity signal (*d*θ^r^). With these power-law transducers the empirical M/PR provides an excellent account for the perceived depth from motion parallax within the range of the variables tested in the head-stationary conditions here.

The actual transducer exponents derived from this experiment are quite interesting and maybe a little confusing. First, the pursuit exponent (e) is smaller than the retinal image motion transducer (r). This is in general agreement with the comparative sizes of the transducers found by Turano and Massof (2001; Table 2) and Freeman (2001; Figure 12). In the current study the relative size indicates that changes in retinal image motion (*d*θ) have a larger effect on changes in perceived depth than changes in pursuit velocity (*d*α). This corresponds to the relative slopes of the lines in Figure 6 (changes in *d*θ) and Figure 7 (changes in *d*α), and the relative slopes of the rainbow and green surfaces along the Log(*d*θ) and Log(*d*α) axes in Figure 9.

However, these transducer values, *e* = 0.192 and *r* = 0.416, are smaller than those that characterize the perception of motion during eye movements, which are typically near 1 [e.g., Figure 12, (Freeman, 2001); although these values are very similar to those for the ill-fitting nonlinear model of (Turano and Massof, 2001), Table 2]. A smaller transducer value means that the visual system is registering, or using, a smaller internal representation of the external physical stimulus. While small transducer values might be problematic for motion perception, the perceptual situation for motion parallax is much different. In the former the mechanism is operating to determine relative velocity, while in the latter the mechanism is determining relative depths. Additionally, with motion parallax the objects are not perceived as moving, but are perceived as stationary within the environment. Therefore, it is, perhaps, not unusual that the different mechanisms operate with different types of inputs. And, perhaps, the lower transducer values contribute to this perceptual difference in object motion with motion parallax. Of course, it is unclear exactly where these signals become inaccurate. Given the higher transducer values for motion perception, it is likely that the reduced transducer values reflect processing of these signals within the mechanism that does the combination for motion parallax.

Finally, the scaling constant applied to the ratio (0.0313) appears to be only related to the chosen units used to represent angles (degrees vs. radians). Recall that Newton's Law of motion says acceleration is proportional to force. The constant of proportionality (mass) depends on units. For instance, if visual angle had been computed in radians, similar to the distance-square approximation for binocular disparity (e.g., Cormack and Fox, 1985) instead of degrees, the scaling constant would be 0.0778 while the transducer values remain unchanged. (A change of scale by a constant (*c*) changes dθ^0.416^/dα^0.192^ by the factor c^0.416^/c^0.192^ and 0.0313372/(c^0.416^/c^0.192^) = 0.0777579 when *c* = π/180. To give the scaling constant a value of 1 in the empirical model, the units of visual angle would have to be represented in a unit equivalent to 5.004 × 10^6^ degrees).

Another curious feature of our empirically measured law is the difference in the two exponents. While these are remarkably similar to the differences observed in the transducer model experiments mentioned above, and while the smaller exponent for pursuit, dα^0.192^, accounts for the foreshortened depth perception and points more strongly to pursuit as the cause of foreshortening, there is another possible contribution to the difference. The brain combines the retinal motion and eye pursuit signals. The mathematical values of motion are much smaller than the mathematical values of the pursuit rates, but the neural representations of these signals could conceivably be scaled differently before making this combination. Their internal units might be different. A scaled combination of logarithmic signals would mathematically be constants in a difference of logs like the least-squares-log formula above (those constants are our transducer exponents). A better understanding of the difference in transducer exponents may reveal insights about the internal neural mechanisms used to recover relative depth from motion parallax.

As mentioned earlier, and addressed with the control conditions, the accuracy and robustness of the empirical motion/pursuit ratio (Equation 4) may depend on how well-depth constancy was preserved in the binocular disparity stimuli with which the motion parallax stimuli were compared. While the Materials and Methods Section outlined the stimulus considerations used to optimize depth constancy in the current study, and the control conditions demonstrated excellent depth constancy, here we consider the implications if depth from binocular disparity were independently overestimated at the near viewing distances used in the current study (Johnston et al., 1994). Hypothetically, with the depth matching procedure used here, this would produce an underestimate the perceived depth from motion parallax from the motion parallax stimulus parameters. Moreover, we can estimate the effect of a hypothetical distortion found with binocular disparity. For this we used the well-known, and often cited, example of distortion provided by Johnston (1991). Using the data extrapolated from her Figure 4 at the two shortest viewing distances (*f* = 53.4 and 107 cm) for both observers (EBJ and JSM), we determined a least squares function of viewing distance. Johnston does not include data at 36 cm, so we needed to extrapolate her result down to our data range. This function was used to scale the depth magnitude estimates at each the viewing distances in the current study (*f* = 36, 54, and 72 cm) with the scaling factor:

(5) $$\frac{d(perceived)}{d(veridical)}=2.015-0.011f$$

Notice that this ratio, *d*(*perceived*) = *d*(*veridical*), is 1 at *f* = 96.2 cm, rather than at the 80 cm viewing distance (e.g., Johnston et al., 1994) that Johnston found by another approach. This increases our depth magnitude estimates at all viewing distances (*f* = 72, 54 and 36 cm) with a greater increase in distortion at smaller f. Compared to the PSE and σ values for the depth discrimination from binocular disparity determined in the control studies, this distortion represents a PSE shift of about 2-to-3 σ values.

With the scaling function given in Equation (5) representing a hypothetical distortion in the perception of depth from binocular disparity, the transducer values found in Equation (4) changed from *r* = 0.416 and *e* = 0.192 to adjusted values of *r*_*a*_ = 0.428 and *e*_*a*_ = 0.148, giving an adjusted empirical motion/pursuit ratio:

(6) $$d_{mp}=\frac{\text{d}\theta^{\text{0}.\text{428}}}{d\alpha^{0.148}}\left(0.0444\right)f$$

Graphically, this hypothetical adjustment would shift the green surface in Figure 9 vertically up by less than half a natural log unit. In units of perceived depth, this adjustment corresponds to a increase in the magnitude of perceived depth from motion parallax of a few to several mm in the parameter space studied here. Interactive graphs of both the adjusted and un-adjusted plots (Figure 9) are included in the Supplementary Material. The reader can move the figures around and see the comparison both with each other and with the motion/pursuit ratio. The data and programs that generated the plots are also included. Of course, this extrapolated adjustment corresponds to an extreme case, but it persuasively demonstrates that any failure of depth constancy with the binocular disparity stimuli would have only a small effect on the interpretation of these results. This is because the documented distortions in the perception of depth from binocular disparity are small compared to the systematic distortion in the perception of depth from motion parallax expressed in the empirical motion/pursuit ratio (Equation 4).

The results of this study show that the M/PR, with the application of a single set of non-linear transducers that represent the inherent inaccuracies of the internal motion and pursuit signals, can account for the perception of depth from motion parallax over a variety of pursuit velocities, retinal image velocities, and viewing distances. Moreover, the empirical M/PR espoused here provides testable, quantitative predictions for parameters outside this range. While the non-linearities suggest the empirical M/PR may generalize to a much wider range of parameters, it is unclear what may happen at very large viewing distances. While the retinal motion and pursuit are subject to a “speed multiplier” effect for long viewing distances while the observer is translating at a higher speed (Nawrot and Stroyan, 2009), the perception of depth may be more closely tied to the apparent distance, rather than the actual physical distance, as it is for stereoscopic depth perception (Cormack, 1984). This would, of course, present an obvious difficulty for the quantitative predictions of the model.

Another important caveat is the empirical M/PR does not account for conditions in which the observer is accelerating and producing involuntary tVOR eye movements. These include conditions in which the observer's head is being translated from side-to-side. In these conditions the compensatory eye movement is a combination of tVOR and smooth pursuit (Miles, 1993, 1998), but it is only the pursuit component of the compensatory eye movement that contributes to the internal signal *d*α (Nawrot and Joyce, 2006). As illustrated by the head-translating conditions in the current experiment, a high velocity eye movement during head translation produces the same *d*_*mp*_ depth magnitude as a slower velocity eye movement with a stationary head. The difference is due to the tVOR contributing to the eye movement gain, but not to the mechanisms responsible for perceived depth.

The results of this study indicate large depth foreshortening with motion parallax. This is found with both head-stationary viewing (which isolates pursuit eye movements) and head-moving conditions (which elicits both pursuit and tVOR eye movements). The empirical M/PR now addresses this depth foreshortening with power-law transducers adjusting the retinal motion (*d*θ) and pursuit (*d*α) signals. The use of power-law transducers here is similar to their use in explaining the inaccuracies in perceived motion during eye movements (Freeman, 2001; Turano and Massof, 2001). However, the exponents found here, for the perception of depth from motion and eye movements, are smaller than those for the perception of motion, but not depth, during eye movements. A possible link between these might be to determine the power-law transducers that model the perception of motion for objects nearer or farther than the fixation plane. Such work would reveal much about how we recover the relative depth for non-fixated moving objects while the observer is also moving, a common occurrence in our cluttered environment.

### Conflict of interest statement

The authors declare that the research was conducted in the absence of any commercial or financial relationships that could be construed as a potential conflict of interest.

## Supplementary material

Supplementary materials present interactive versions of the data presented in Figure 8, along with the effects of a hypothetical distortion in the perception of depth from binocular disparity presented in the discussion. The file is in Mathematica CDF format, which requires the free Mathematica CDF reader available at (http://www.wolfram.com/cdf-player/).
