# Supplementary material for: Modeling depth from motion parallax with the motion/pursuit ratio
Source: Front Psychol. 2014 Oct 6;5:1103. doi: 10.3389/fpsyg.2014.01103 (PMC4186274; doi:10.3389/fpsyg.2014.01103)
Supplement: Supplementary file 2 [file Presentation1.ZIP › Supplement/SupplementNawrotEtAl.html]

xml version="1.0" encoding="UTF-8"?


Untitled


Supplementary Interactive Figures for

Modeling depth from motion parallax with the motion/pursuit ratio

by Mark Nawrot, Michael Ratzlaff, Zachary Leonard and Keith Stroyan

The Empirical Motion/Pursuit Law by Log-Least-Squares

Geometrically,   (for small ratios)

Taking (natural) logs of both sides gives the log-linear relation we expect:

so we use the variables logdθ, logdα, and logd/f and plot our data with a least squares fit,

graphed next.

The Figure can be moved by putting the mouse on the graph, clicking and dragging.

Figure S1: Log-Log-Log Plot of M/PR (Formula 2) (gray), Least Sqaures Fit (green), Data (rainbow) with lines of constant perceived relative depth

Depth-adjusted Data

Johnston 1991 observed overestimates of stereo depth perception that increase with closer fixate distance.  Extrapolating those results and applying the scaling to our data gives the data shown in tan shades with least squares fit shown as the blue plane.

The Figure can be moved by putting the mouse on the graph, clicking and dragging.

Figure S2: Log Plots of:  M/PR (Formula 2) (gray), Adjusted Data (tan shades), Adjusted Least Squares Fit (blue), UnAdjusted Least Squares Fit (green)

Algebraic Comparison

Adjusted empirical M/P Law:

Un-Adjusted empirical M/P Law: 0.0313

Comparison at Expanded Vertical Scale

Figure S3: Log Plots of Data Plot with Least Squares and Johnston 1991 Extrapolated Adjusted Data and Least Squares Fit

UnAdjusted Least Sqaures Fit (green), UnAdjusted Data (rainbow) with lines of constant perceived relative depth

Adjusted Least Sqaures Fit (blue), Adjusted Data (tan shades) with lines of constant perceived relative depth

Figure S4: Combined Log Plots of  Data Plot and Johnston 1991 Extrapolated Adjusted Data and Least Squares Fit

The Figure can be moved by putting the mouse on the graph, clicking and dragging.

Note: The Mathematica programs that generate these figures are in the closed cells of the download.

Created with the Wolfram Language
