# Supplementary figures and images for: Modeling depth from motion parallax with the motion/pursuit ratio
Source: Front Psychol. 2014 Oct 6;5:1103. doi: 10.3389/fpsyg.2014.01103 (PMC4186274; doi:10.3389/fpsyg.2014.01103)

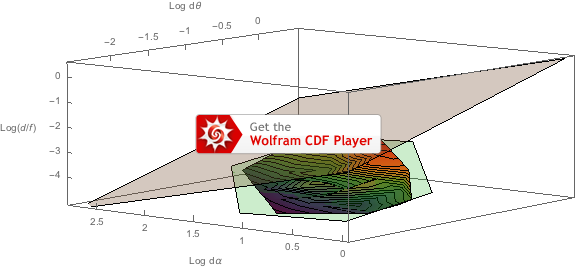

Supplement: Supplementary file 2 [file Presentation1.ZIP › Supplement/FigureS1.png]

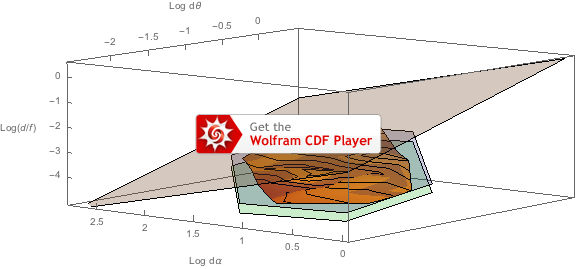

Supplement: Supplementary file 2 [file Presentation1.ZIP › Supplement/FigureS2.png]

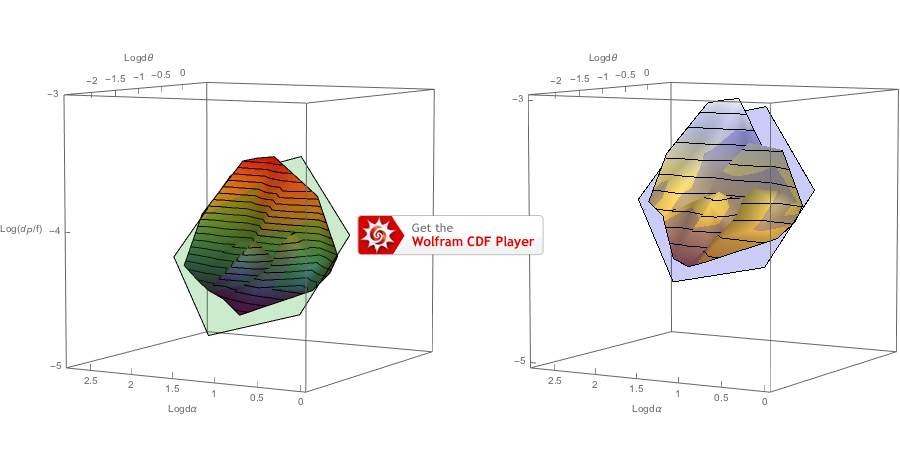

Supplement: Supplementary file 2 [file Presentation1.ZIP › Supplement/FigureS3.png]

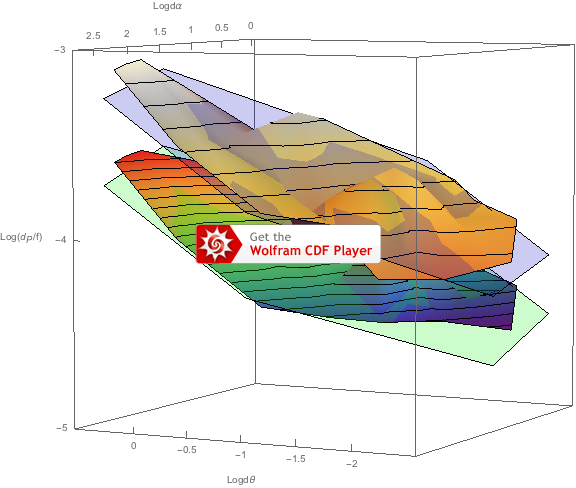

Supplement: Supplementary file 2 [file Presentation1.ZIP › Supplement/FigureS4.png]

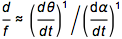

Supplement: Supplementary file 2 [file Presentation1.ZIP › Supplement/HTMLFiles/SupplementNawrotEtAl_1.png]

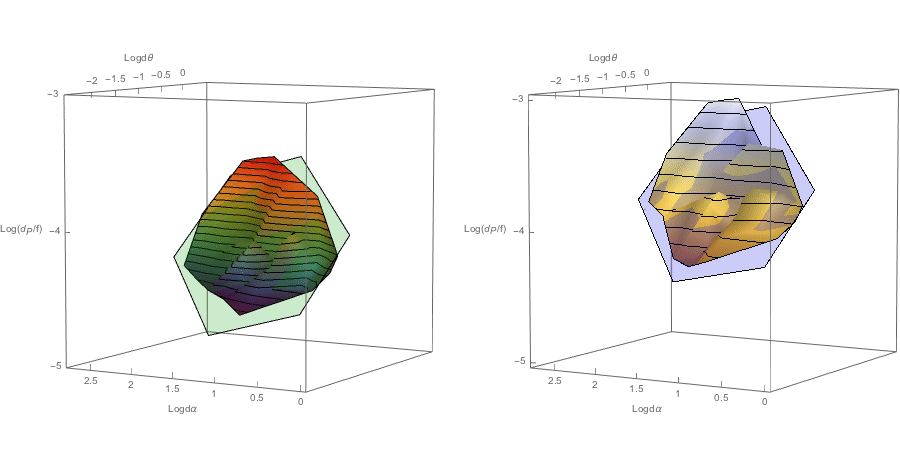

Supplement: Supplementary file 2 [file Presentation1.ZIP › Supplement/HTMLFiles/SupplementNawrotEtAl_10.gif]

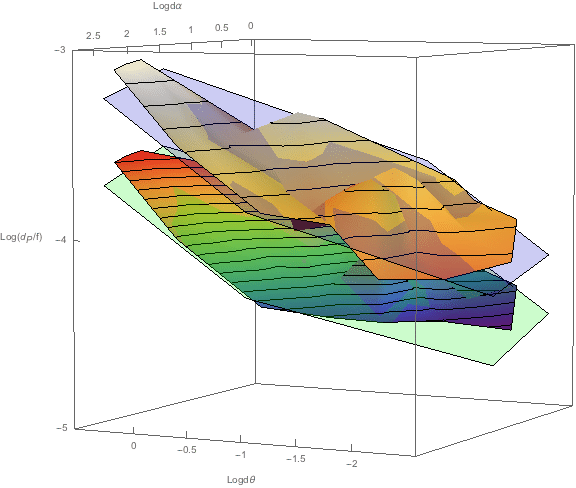

Supplement: Supplementary file 2 [file Presentation1.ZIP › Supplement/HTMLFiles/SupplementNawrotEtAl_11.gif]

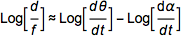

Supplement: Supplementary file 2 [file Presentation1.ZIP › Supplement/HTMLFiles/SupplementNawrotEtAl_2.png]

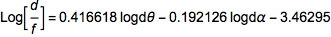

Supplement: Supplementary file 2 [file Presentation1.ZIP › Supplement/HTMLFiles/SupplementNawrotEtAl_3.png]

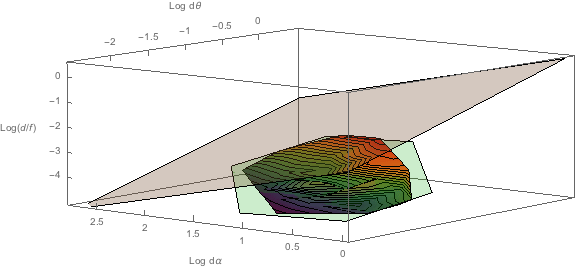

Supplement: Supplementary file 2 [file Presentation1.ZIP › Supplement/HTMLFiles/SupplementNawrotEtAl_4.gif]

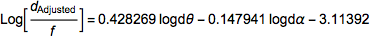

Supplement: Supplementary file 2 [file Presentation1.ZIP › Supplement/HTMLFiles/SupplementNawrotEtAl_5.png]

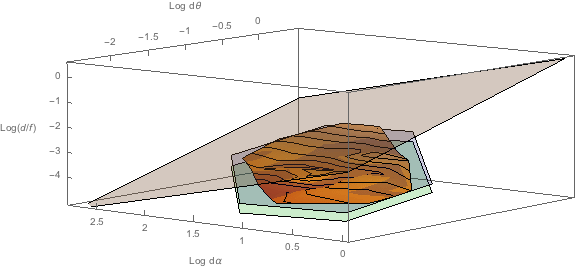

Supplement: Supplementary file 2 [file Presentation1.ZIP › Supplement/HTMLFiles/SupplementNawrotEtAl_6.gif]

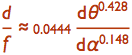

Supplement: Supplementary file 2 [file Presentation1.ZIP › Supplement/HTMLFiles/SupplementNawrotEtAl_7.png]

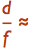

Supplement: Supplementary file 2 [file Presentation1.ZIP › Supplement/HTMLFiles/SupplementNawrotEtAl_8.png]

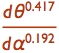

Supplement: Supplementary file 2 [file Presentation1.ZIP › Supplement/HTMLFiles/SupplementNawrotEtAl_9.png]
